# Articulating Video Stylets in the Setting of Simulated Traumatic Cervical Spine Injury: A Comparison with Four Other Devices and Approaches to Endotracheal Intubation

**DOI:** 10.3390/jcm13247760

**Published:** 2024-12-19

**Authors:** Federica Merola, Simone Messina, Cristina Santonocito, Marco Sanfilippo, Giulia Sanfilippo, Federica Lombardo, Giovanni Schembari, Paolo Murabito, Francesca Rubulotta, Filippo Sanfilippo

**Affiliations:** 1 Department of Anaesthesia and Intensive Care, Policlinico-San Marco University Hospital, Via S. Sofia n 78, 95123 Catania, Italy; merolafede@gmail.com (F.M.); messina.simone05@gmail.com (S.M.); cristina.santonocito@gmail.com (C.S.); lombardofederica92@gmail.com (F.L.); giovannischembari49@gmail.com (G.S.); paolomurabito@gmail.com (P.M.); frubulotta@hotmail.com (F.R.); 2 School of Anesthesia and Intensive Care, University “Magna Graecia”, 88100 Catanzaro, Italy; marco.sanfilippo.96@gmail.com (M.S.); giulia.snf@gmail.com (G.S.); 3 Section of Anesthesia, Department of General Surgery and Medical-Surgical Specialties, University of Catania, 95124 Catania, Italy

**Keywords:** direct laryngoscopy, endotracheal intubation, fiberoptic bronchoscopy, manikin, video laryngoscopy

## Abstract

**Background:** Simulation offers the opportunity to train healthcare professionals in complex scenarios, such as those with as traumatized patients. **Methods:** We conducted an observational cross-sectional research simulating trauma with cervical immobilization. We compared five techniques/devices: direct laryngoscopy (DL), videolaryngoscopy (VLS, Glidescope or McGrath), combined laryngo-bronchoscopy intubation (CLBI) and articulating video stylet (ProVu). The primary outcomes were as follows: (1) success rate (SR) by third attempt (each lasting up to 60 s), and (2) corrected time-to-intubation (cTTI, accounting for failed attempts). **Results:** In a single center, we enrolled 42 consultants experienced in DL/VLS, but reporting no experience with ProVu, and hypothesized that ProVu would have offered encouraging performances. By the third attempt, ProVu had a SR of 73.8%, identical to Glidescope (*p* = 1.00) and inferior only to McGrath (97.6%; *p* = 0.003). The cTTI (seconds) of ProVu (57.5 [45–174]) was similar to Glidescope (51.2 [29–159]; *p* = 0.391), inferior to DL and McGrath (31.0 [22–46]; *p* = 0.001; and 49.6 [27–88]; *p* = 0.014, respectively), and superior to CLBI (157.5 [41–180]; *p* = 0.023). Conclusions: In consultants with no experience, as compared to DL and VLS, the video stylet ProVu showed encouraging results under simulated circumstances of cervical immobilization. The results should be interpreted in light of the participants being novices to ProVu and skilled in DL/VLS. Adequate training is required before the clinical introduction of any airway device.

## 1. Introduction

Endotracheal intubation (ETI) is a fundamental skill for anesthesiologists, but the approach profoundly differs between elective contexts without anticipated difficulties to more complex situations, such as during emergencies. Several reasons may render airway management challenging during an emergency; this is typical of patients admitted with polytrauma, where together with possible unstable cardiovascular and respiratory conditions and the uncertainty regarding patient’s fasting, the cervical collar further challenges airway management, limiting the neck mobility and mouth opening [1]. In this challenging context, ETI should take the extra care of not creating, nor exacerbating, a neurological injury [2]. Nonetheless, any delay in oxygenating the patient may have catastrophic consequences, for instance with secondary brain injury due to hypoxia. Hence, the staff readiness and exercise in performing ETI under such complex scenarios should not be underestimated. In this regard, regular training with simulation seems the most practical option [3,4,5,6]. Furthermore, simulation sessions represent a great opportunity for gaining confidence, including confidence with alternative devices and techniques for airway management [7,8].

As the neck position and the mouth opening are not grossly modifiable in the case of polytrauma, the choice of device can be crucial. Whilst the direct laryngoscopy (DL) is certainly the most used maneuver, the video-laryngoscope (VLS) has recently gained momentum with randomized trials showing its greater performances in airway management [9,10,11,12]; moreover, a meta-analysis of trials enrolling adults patients showed that VLS was associated with fewer failed ETI attempts and complications [13]. The latest guidelines for the management of the airways in victims of trauma with suspected or established cervical spine injury suggest the use of VLS [2]. This is not surprising, as VLSs not only improve the glottis visualization but these devices may also ensure a greater stability in the cervical spine during the procedure, [14] with recently developed VLS that appears promising [15]. Notably, the learning curve of VLS appears to be faster than the DL technique, especially for beginners [16,17,18,19]. However, the main limitation of VLS remains the challenge of adequately directing the endotracheal tube through the glottis; consequently, the use of a stylet is usually recommended [20]. An alternative approach, the so-called combined laryngo-bronchoscopy intubation (CLBI), has been proposed and entails the introduction of a fiber-optic bronchoscope (FOB) with the partial help of a DL (or a VLS) to displace the tongue and widen the upper pharynx. Notably, promising results for the CLBI approach have been described by simulation studies [21,22], and reported in complex clinical scenarios [23,24,25], even in the pediatric population [26]. Finally, a novel and interesting alternative could be the use of the newer flexible video stylets, the performances of which have been improved by the recent technical developments (i.e., high-resolution camera, wide view, anti-fog coating). In patients with polytrauma, flexible video stylets may have comparable advantages to the CLBI technique (wide range of movements), but with the value of using a single and smaller device, with a design and a handling more similar to the DL or VLS than to FOB.

In the belief that the flexible video stylets could be very useful in the polytrauma setting, we performed a manikin study simulating an airway scenario of polytrauma with a cervical collar in place. In a population of consultants without previous experience with flexible video stylets, we compared their performances with those of DL, VLS, and CLBI. Our hypothesis was that when compared to DL and VLS, the flexible video stylets would show similar performances by the third ETI attempt, and also improved performances in respect to the CLBI approach.

## 2. Materials and Methods

A cross-sectional observational trial was conducted at the “*Cristian Ilardi*” Simulation Center based at “Policlinico G. Rodolico—San Marco” University Hospital, Catania (Italy). The study participants were consultants with a diploma in anesthesia, critical care and pain working at our institution. As baseline variables of the participating population, we registered their demographics (gender and age), the year when their anesthesiological training was completed, and their confidence with the airway devices and approaches tested in the study. The clinical experience of each participant was evaluated by the self-estimation of the number of previous ETIs performed with each device/technique.

Regarding the latter, we studied five approaches to ETI in a simulated airway scenario of cervical immobilization as it typically happens in patients with suspected cervical trauma. The manikin used for the study was Larry Intubation Trainer (Armstrong Medical Inc., 575 Knightsbridge Parkway, P.O. Box 700, Lincolnshire, IL 60069-0700, USA), placed on a board with adjustable height. The height by default was set at the xiphoid process of each attending anesthesiologist, though we changed it to their preference. Cervical immobilization was obtained applying a cervical collar.

**Figure 1:**
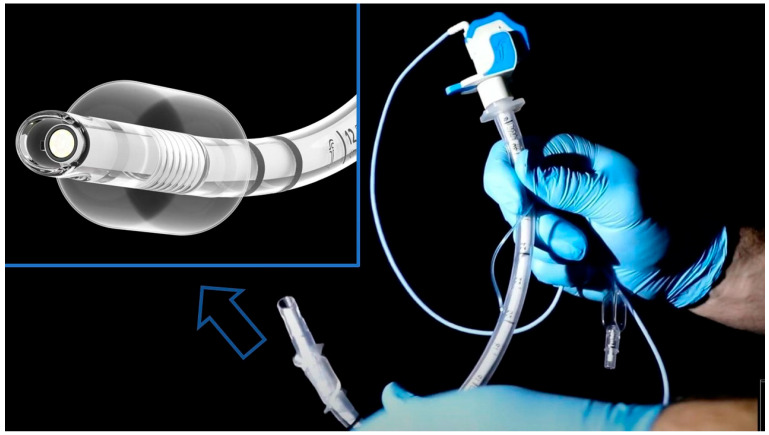
The articulating flexible video stylet ProVu^®^ used for the present simulation study. A detail of the flexible tip with a camera is shown in the top left corner.

Four independent authors executed the simulation session (SM, MS, GS, FL), providing a standardized 10 min teaching session before the start of the study to explain the study methods and devices to the participants. The four operators used a chronometer for time recording and registered the overall study data on a password-protected Excel spreadsheet. During sessions, the consultants were not allowed to stand during the attempts from other participants, so that this would not influence the results producing biases. As mentioned, five approaches were studied with a randomized order of device, obtained through the use of sealed envelopes: (1) DL, using Macintosh laryngoscope equipped with a blade size 3 (Mercury Medical, Clearwater, FL, USA); (2) a VLS with the monitor mounted on device (McGrath) equipped with MAC blade X3 (McGrath; Aircraft Medical Ltd., Edinburgh, UK); (3) VLS with screen set apart from the device (Glidescope, Verathon Inc., 20001 North Creek Parkway, Bothell, WA 98011, USA); (4) an articulating flexible video stylet (ProVu^®^ Video stylet, Flexicare Inc., Irvine, CA, USA) introduced in the upper airways with the assistance of DL; (5) a CLBI approach, using a DL and a disposable bronchoscope (aScope™ size 4, Ambu A/S Baltorpbakken 13, DK-2750, Ballerup, Denmark). The articulating flexible video stylet ProVu^®^ is shown in Figure 1 with a detail of the flexible tip with camera. The present study was not financially supported. The airway devices were accessible from our Simulation Center where we regularly train residents, apart from the video stylet that was provided by a local company with no role nor influence in any part of this research study. In order to avoid waste or products and production of carbon footprint; a device was substituted only if became damaged.

The ETI were performed with a lubricated endotracheal tube with an internal diameter 7.5 mm, and a stylet was available for the DL upon request, whilst with the VLS techniques a semi-rigid stylet was preventatively loaded on the endotracheal tube. Every so often, we used lubricant to wet the manikin and endotracheal tube. The study team started the chronometer count when the participant took the airway device; time recording was terminated when the operator declared ETI.

We selected two primary outcomes consistent with our previous simulation studies: the success rate (SR) in achieving ETI, and the corrected time to intubation (cTTI). Our methods allowed a maximum of three ETI attempts for each device/technique. A success was confirmed by the research team following lung insufflations. Esophageal intubation or attempts lasting over 60 s were declared as a failure. The cTTI is defined as the time to achieve intubation corrected for the number of attempts, meaning that for each failure before achieving successful ETI, an extra 60 s was counted (i.e., a successful ETI obtained at 35 s after one previous failure attempt ends up in a count of 95 s of cTTI). If the participant failed all the three attempts, a count of 180 s was imputed. Secondary endpoints were the time to intubation that was not corrected for failed attempts (uncorrected, uTTI), the time to ventilation corrected for failed attempts (cTTV, time recording terminated at successful bag inflation), ease of intubation as rated by participants with a Likert scale (1–10, with 10 meaning very easy). We registered data on the glottic view as per the Cormack–Lehane grade [27] and the Percentage of Glottis Opening (POGO) [28] (0–100%). Though Cormack–Lehane and POGO are validated for DL and VLS only, we asked participants to report such visualization also for CLBI and ProVu techniques.

Data distribution was assessed with Shapiro–Wilk test. Data are reported as percentages/numbers (categorical variables) and analysis conducted with Fisher’s exact test [29]. Continuous variables are reported as mean ± standard deviation (for the data with normal distribution), or as median and interquartile range [IQR] (when data were not normally distributed), and analyzed with the paired test (the t-Student or the Wilcoxon’s rank test). We considered significant differences as instances where the *p* value was <0.05.

## 3. Results

In total, we enrolled 42 participants in this study in the period May–June 2023. The characteristics of the volunteers were 46.5 ± 8.4 years-old, 52.4% females (n = 22), 15 ± 9 years of experience after completing their anesthesiological training. None of them reported that they had received special training in difficult airway and cervical spine immobilization.

On a scale from 0 to 10, the self-reported experience with each device/technique for airway management was as follows: (1) DL 10 [10]; (2) Glidescope 8 [5–10]; (3) McGrath 7 [3–9]; (4) CLBI 2 [0–6]; (5) ProVu 0 [0, 1]. The performances of each device are reported in Table 1, describing SR at the 1st, 2nd and 3rd attempt, as well as the cTTI and uTTI.

The paired statistical analyses comparing performance of the five techniques/approaches for airway management in terms of the SR by the third attempt are shown together with their findings on cTTI in Table 2.

In Table 3, we report the findings on the operator judgement on the ease of use as well as the POGO and the Cormack–Lehane evaluations for all the devices/techniques.

## 4. Discussion

The present study pairs with an investigation that our group previously performed on a simulation setting of normal airway conditions [30]. In both studies, we tested the performances of a novel flexible video stylet (ProVu) with those of more conventional airway devices (DL and VLS), as well as with the combined technique embracing the use of FOB and DL (CLBI). As for the previous study, the present investigation was conducted with 42 participating anesthesiologists of a single institution. Hereby, we present results obtained from a scenario of increased difficulty due to cervical immobilization with a rigid collar, a condition typically encountered in patients with polytraumas.

To our knowledge, we conducted the largest study testing the novel flexible video stylet ProVu. Before discussing our results, we believe it is of utmost importance that readers keep in their minds the different levels of confidence and experience that were self-reported by the operators with each of the devices and techniques tested. Indeed, as shown in Table 3, the operators had great confidence with DL and the two VLSs (Glidescope and McGrath), very low confidence with the CLBI, and no knowledge at all on ProVu. Indeed, the operator’s knowledge on the use of ProVu remained just limited to the information provided during our standardized 10 min teaching session before the begin of the study. Therefore, on reflection, our hypothesis regarding similar performances by the 3rd attempt by the flexible video stylet as compared to DL and VLS was probably too optimistic. In truth, the flexible video stylet performed well in securing ETI, since it showed similar SR to all the other devices, apart from the DL (at the 1st attempt) and the McGrath (at the 3rd attempt). The latter two devices (DL and McGrath) were also performing superiorly to the flexible video stylet in terms of cTTI; however, for this outcome the flexible video stylet had similar performances to the Glidescope and superior to the CLBI, partially in line with our preliminary hypothesis. Indeed, we also hypothesized that the flexible video stylet would have been superior to the CLBI approach, which was particularly confirmed by the lower cTTI (57.5 s [45–174] as compared to 157.5 s [41–180] for the CLBI; *p* = 0.023).

We believe that our results are encouraging for the future implementation of the flexible video stylets. However, before any clinical implementation of the ProVu (or of any other airway device), adequate training in simulated and/or elective conditions is required, so that operators obtain expertise and feel confident with the device. The latest devices developed of this category have been improved by the presence of a high-resolution camera and large field of view, and in some instances by anti-fog coating. Additionally, the flexible rod can be detached or pulled back if the operator needs more tip flexibility, such as during ETI from the nostrils or when intubation is performed via laryngeal mask. Further, it is important to note that movements of some video stylets have been potentiated, and in some devices this range becomes greater than 90°. Therefore, flexible video stylets, due to their maneuverability, could represent a promising option. The maneuverable tip of the Provu enables the operator to pass the video stylet and the endotracheal tube into the trachea with a direct view of the carina, minimizing the risks of trauma [31].

The latest guidelines for the airway management in patients with suspected or confirmed cervical spine injury suggest the use of VLS whenever possible for ETI in these patients, though they are unable to recommend a particular type of VLS or specific type of blade [2]. Our study was designed in the belief that in the polytrauma setting, the flexible video stylet may be a valid alternative to the use of DL or VLS. It is known that the flexible video stylet approach provides advantages when approaching ETI for patients with limited cervical motion [32]. Indeed, flexible video stylets have the same advantages ensured by the CLBI technique (wide range of movements to direct the endotracheal tube) with the additional value of using a single and smaller device. Their characteristics could be invaluable in a crowded scenario of a patient admitted with polytrauma where several clinical professionals are involved in a limited space and with the need to act rapidly and to perform several procedures (i.e., chest drain insertion, echocardiography). The handling of the flexible video stylet is more similar to the manipulation of the DL or VLS, which may explain the better results of the flexible video stylet as compared to the CLBI. In a complex setting with a cervical collar, such as in patients admitted to hospital with polytrauma, the flexible video stylet may allow ETI without any aid from DL/VLS which may result in minimizing neck movements. In this regard, the flexible video stylet represents the only device/technique, among those we have tested, that could be safely introduced with no effects on displacement of the temporo-mandibular joint as it does not require any energy delivered from the operator.

The airway management in a polytrauma patient should be always considered difficult, at least because of the suspected cervical spine injury and consequent positioning of a cervical collar. Cervical stabilization is usually obtained by positioning a rigid cervical collar, which in turn reduces the patient’s mouth opening, making the introduction of a laryngoscope more challenging. Moreover, the collar itself also causes anterior displacement of the chin and larynx, further complicating the ETI procedure [1].

Unlike the elective scenario, with adequate time to plan airway management, the polytrauma context is often much more complex. Indeed, constraints are present not only because of the cervical collar, but also due to the need to rapidly ensure airway control and provide adequate oxygen delivery in unstable patients [33,34]. As hemodynamic conditions may be severely compromised in patients with polytrauma for several reasons (bleeding, vasoplegia, pneumothorax, cardiac contusion, etc.), the possibility to intubate without the use of a laryngoscope, but rather with a device that could require a lower use of sedatives should be favorably considered for the potentially smoother hemodynamic impact. Of course, a manikin study cannot evaluate and weigh the sympathetic stimulation due to the laryngoscopy (whether it is a DL or VLS) nor can it gauge the advantages of reducing sedative agents when using a flexible video stylet. More clinical research is certainly needed to understand the advantages of flexible video stylets in different clinical settings, from the anticipated difficult airways to emergency conditions.

Finally, it must be noted that this study represents a “plastic on” trial where we use devices responsible for carbon footprint. We are aware of the importance of the greenhouse effect and the need to reduce carbon dioxide production [35], where healthcare systems contribute to 5–10% of worldwide emissions [36]. In this regard, the operating rooms consume a high proportion of disposables generating waste products [37], with a gap in the implementation of clinical approaches to improve sustainability in the operating room [38]. A move towards green anesthesia is certainly needed, and does not seem to compromise patient’s safety [39]. For instance, in a model of over 17.000 ETI/year, it was estimated that the use of reusable as compared to single-use DL blades may save over 26 tons of carbon dioxide equivalents, almost 7 tons of oil equivalents, producing an average economical saving of 0.35 EUR/ETI [40]. For such reasons, both the pre-clinical research investigating devices via simulation studies, and subsequently the clinical decision to introduce any device, should consider the environmental impact. In light of these considerations, in order to minimize plastic waste, we substituted a device or a tube only if it was needed because of damage.

### Strengths and Limitations

Our study has some strengths but also several limitations that should be discussed. The main strength is that we focused on a difficult airway scenario frequently encountered by clinicians, and that the study is among the largest studies evaluating the performance of the novel flexible video stylet ProVu. A second strength of the study is the statistical approach where we adopted the cTTI, which accounts for the previously unsuccessful attempts to intubate. This choice was in line with our previous research [30] and it is based on the consideration that any failed attempt to intubate exposes the patients to potentially life-threatening risks, even more so in the setting an unstable patient with difficult airways, as frequently happens in polytrauma. This methodology avoids the fact that any technique ensuring short TTI but poor SR in the first attempt may be considered as outpacing other approaches with much higher SR but slightly longer TTI. Whether this approach is accepted or not, we have described it clearly, whilst several studies have been ambiguous in the way they handled the failed attempts. Moreover, we also described the results of the uTTI (not accounting for unsuccessful attempts), which were mostly superimposable to those obtained with the correction for the SR (cTTI). A third strength of our study is probably the randomization in the order of use of each device/technique.

As mentioned, several limitations should be considered in our study. To start with, despite its relatively large sample size, it remains a single-center study and external validation is recommended. Furthermore, the results are conditioned by the absence of skills in the use of ProVu and in the participants’ low levels of experience in CLBI, and could be unbalanced by the fact that some of them work prevalently in the operating room and others more commonly rotate in the intensive care unit. Moreover, we reported the experience with each device stated by each anesthesiologist; conversely, they were not confident in providing a relatively precise estimate in the number of difficult ETI performed. Last but not least, the value of simulation in the setting of airway management cannot be overemphasized. However, despite pooled results from a meta-analysis reinforcing the valuable role of simulation in training the staff in airway management [41], all simulation studies (as ours) suffer from inherent biases. First, a manikin scenario cannot reproduce real-life challenges (i.e., the presence of secretions and bleeding), conditioning the overall performance of each device/technique. In this regard, the devices equipped with cameras (such as VLS, FOB and video stylets) will particularly suffer from blurred or eventually obscured vision. Second, a higher than expected SR has been described in difficult airway simulated scenarios [42], and this can be explained by several factors. Among these, successful attempts may result from blind ETI. Despite guidelines recommending against the blind insertion of the endotracheal tube due to the risk of damage and the low chances of success in the real life [43], during simulation studies participants may try to insert the endotracheal tube as they do not perceive a real risk to harm the patient, nor do they perceived it worsening visibility due to traumatic bleeding. Third, it is not possible to replicate the anxiety of the operator, which may happen in real life when stressful circumstances mount during deteriorating clinical conditions (i.e., occurrence of severe desaturation and/or hypotension) [44]. Fourth, studies conducted in both pediatric and adult manikins have suggested dissimilarities between the manikins and real human anatomy [45,46,47]. There are also other limitations to our study. We did not study the technique of FOB intubation in all its different possibilities, including through a supraglottic airway device. We obviously did not study FOI with an awake patient either, because of the simulation setting. Interestingly, a European survey conducted in 15 countries among anesthesiologists and emergency physicians found that in patients with cervical spinal cord injuries, the FOI was the second most preferred technique for ETI after DL. Moreover, over 50% of respondents considered that FOI caused the least cervical spine movements. Nonetheless, most respondents indicating FOI as the preferred approach were reported as not being “skilled” [48].

## 5. Conclusions

We conducted a single-center observational cross-sectional study in an airway scenario simulating a traumatized patient with cervical immobilization, comparing five techniques/devices. The study was performed in a population of consultants with no prior experience with the articulating video stylet ProVu, and excellent experience in DL and VLS. As compared to DL and VLS, the articulating video stylet ProVu showed encouraging results in simulated airways with the additional challenge of cervical immobilization. In light of the unbalanced experience with devices, the ProVu could be a very valid device and deserves clinical studies.

## Figures and Tables

**Table 1:** The two primary outcomes of the study were the success rate (SR) and the corrected time to intubation (cTTI). Results are presented for the five devices/techniques studied. The success rates (SR, at the 1st, 2nd, 3rd attempt and overall) and the failure rates (by the 3rd attempt) are listed in the top part of the table, followed by the cTTI (together with the uncorrected time to intubation (uTTI). The statistical differences in performance between techniques are provided separately in Table 2. DL: direct laryngoscopy; CLBI: combined laryngo-bronchoscope intubation.

	DL	Glidescope	McGrath	CLBI	ProVu
SR at 1st attempt	35/42 (83.3%)	23/42 (54.8%)	23/42 (54.8%)	15/42 (35.7%)	22/42 (52.4%)
SR at 2nd attempt	+1/42 (2.4%)	+8/42 (19%)	+16/42 (38%)	+5/42 (11.9%)	+7/42 (16.6%)
SR at 3rd attempt	+1/42 (2.4%)	+0/42 (0%)	+2/42 (4.8%)	+5/42 (11.9%)	+2/42 (4.8%)
Overall SR	37/42 (88.1%)	31/42 (73.8%)	41/42 (97.6%)	25/42 (59.5%)	31/42 (73.8%)
Failure rate	5/42 (11.9%)	11/42 (26.2%)	1/42 (2.4%)	17/42 (40.5%)	11/42 (26.2%)
cTTI	31.0 [22–46]	51.2 [29–159]	49.6 [27–88]	157.5 [41–180]	57.5 [45–174]
uTTI	26.2 [22–37]	28.3 [22–35]	28.4 [19–38]	36.4 [33–49]	37.9 [27–50]

**Table 2:** Pair-wise comparison and differences in success rate (SR), and corrected time to intubation (cTTI) between the five techniques that are the subject of this study are listed. DL: direct laryngoscopy; CLBI: combined laryngo-bronchoscope intubation. In bold, we highlight the results that were statistically significant.

cTTI	Glidescope	McGrath	CLBI	ProVu
DL	0.011	0.328	<0.001	0.001
Glidescope		0.095	0.003	0.391
McGrath			<0.001	0.014
CLBI				0.023
**SR at 1st attempt**				
DL	0.009	0.009	<0.001	0.005
Glidescope		1.00	0.124	1.00
McGrath			0.124	1.00
CLBI				0.187
**SR at 3rd attempt**				
DL	0.163	0.202	0.006	0.163
Glidescope		0.003	0.247	1.00
McGrath			<0.001	0.003
CLBI				0.247

**Table 3:** Secondary outcomes. Ease of use, Cormack–Lehane and percentage of glottis opening [POGO]) reported by the 42 operators participating in the study with each of the five devices/techniques. Ease of use was judged on a Likert-scale (1–10). Results are described as median [interquartile range]. DL: direct laryngoscopy; CLBI: combined laryngo-bronchoscope intubation.

Device	Ease of Use	Cormack–Lehane (1, 2a, 2b, 3, 4)	POGO
DL	10 [7–10]	1-1-2-18-20	14 [0–25]
Glidescope	8 [6–10]	5-9-7-16-4	50 [25–75]
McGrath	8 [6–10]	12-6-10-11-2	50 [25–90]
CLBI	5 [2–8]	19-1-1-10-10	37.5 [13.75–100]
Provu	4 [1–7]	12-6-1-12-11	25 [13.75–100]

## Data Availability

Data are available on reasonable request to the corresponding author.
